# Subcellular localization and Egl-mediated transport of telomeric retrotransposon *HeT-A* ribonucleoprotein particles in the *Drosophila* germline and early embryogenesis

**DOI:** 10.1371/journal.pone.0201787

**Published:** 2018-08-29

**Authors:** Maria Kordyukova, Valeriya Morgunova, Ivan Olovnikov, Pavel A. Komarov, Anastasia Mironova, Oxana M. Olenkina, Alla Kalmykova

**Affiliations:** 1 Institute of Molecular Genetics, Russian Academy of Sciences, Moscow, Russia; 2 Department of Biochemistry, Faculty of Biology, Lomonosov Moscow State University, Moscow, Russia; Tulane University Health Sciences Center, UNITED STATES

## Abstract

The study of the telomeric complex in oogenesis and early development is important for understanding the mechanisms which maintain genome integrity. Telomeric transcripts are the key components of the telomeric complex and are essential for regulation of telomere function. We study the biogenesis of transcripts generated by the major *Drosophila* telomere repeat *HeT-A* in oogenesis and early development with disrupted telomeric repeat silencing. In wild type ovaries, *HeT-A* expression is downregulated by the Piwi-interacting RNAs (piRNAs). By repressing piRNA pathway, we show that overexpressed *HeT-A* transcripts interact with their product, RNA-binding protein Gag-*HeT-A*, forming ribonucleoprotein particles (RNPs) during oogenesis and early embryonic development. Moreover, during early stages of oogenesis, in the nuclei of dividing cystoblasts, *HeT-A* RNP form spherical structures, which supposedly represent the retrotransposition complexes participating in telomere elongation. During the later stages of oogenesis, abundant *HeT-A* RNP are detected in the cytoplasm and nuclei of the nurse cells, as well as in the cytoplasm of the oocyte. Further on, we demonstrate that *HeT-A* products co-localize with the transporter protein Egalitarian (Egl) both in wild type ovaries and upon piRNA loss. This finding suggests a role of Egl in the transportation of the *HeT-A* RNP to the oocyte using a dynein motor. Following germline piRNA depletion, abundant maternal *HeT-A* RNP interacts with Egl resulting in ectopic accumulation of Egl close to the centrosomes during the syncytial stage of embryogenesis. Given the essential role of Egl in the proper localization of numerous patterning mRNAs, we suggest that its abnormal localization likely leads to impaired embryonic axis specification typical for piRNA pathway mutants.

## Introduction

Telomeres are DNA-protein complexes that protect the ends of linear eukaryotic chromosomes. In most species telomeric repeats are synthesized by telomerase consisting of reverse transcriptase and an RNA template. Telomere-associated proteins form the telomere protection shelterin complex [1]. Recently it was found that telomeric repeats are transcribed and give rise to long non-coding RNAs, TERRA [2]. TERRA molecules interact with shelterin proteins, telomerase, chromatin remodeling factors and localize near telomeres suggesting a structural role in telomere architecture [3]. However, TERRA may also bind and regulate different genic targets indicating its role as an epigenetic factor in gene expression [4]. Moreover, the large network of TERRA interacting proteins suggests that TERRA plays a significant role not only in telomere regulation but also in various cellular pathways [4, 5]. However, the functional significance of most interactions between TERRA and its partners remains poorly understood.

Using *Drosophila* we conducted a systematic study of the biogenesis and function of telomeric transcripts and their associated proteins in the female germline and in early development. Telomerase was not found in the *Drosophila* genome [6]. A unique feature of *Drosophila* telomeres is that they are composed of LINE (long interspersed nuclear element) retrotransposons; namely, *HeT-A*, *TART* and *TAHRE*, among which *HeT-A* is the most abundant [7]. Despite the different nature of telomeric repeats between *Drosophila* and species encoding telomerase, the basic mechanisms of telomere maintenance are similar [8]. *Drosophila* telomeric protein complex is structurally different from human shelterin, but is functionally analogous and protects the chromosome ends from degradation and fusion [9, 10]. Transcription of telomeric repeats is a conserved feature described in all studied species. Telomeric transcripts in *Drosophila* were described a decade earlier than human TERRA [11, 12]. The *Drosophila* telomeric transcriptome in the germline consists of both long retrotransposon transcripts and small RNAs [13, 14]. *HeT-A* and *TART* produce multiple sense and antisense transcripts, the latter containing multiple introns [14–16]. Unusual regulatory regions of *HeT-A* and *TART* retrotransposons drive the bidirectional transcription [16, 17]. *HeT-A* sense transcripts may be considered as functional analogues of both the telomerase RNA template and TERRA RNA. Clearly, the expression of telomeric elements must be strictly controlled to ensure regulation of telomere length. In *Drosophila* ovaries, such control is mediated by a distinct mechanism of RNA interference, the piRNA (PIWI interacting RNA) pathway. In the germline, antisense transcripts of telomeric retrotransposons serve as piRNA precursors, regulating the abundance of sense coding transcripts [13, 14]. In the presence of piRNAs, the expression of telomeric repeats is repressed, while mutations in piRNA pathway genes lead to a drastic accumulation of telomeric transcripts and an increased rate of heritable terminal retrotranspositions [13]. piRNA pathway disruption strongly affects telomeric chromatin state and *HeT-A* expression in the germline leading to severe developmental defects [18–21].

Telomeres have certain features of heterochromatin, therefore, their transcriptional activity in a normal cell is repressed. Increased level of telomere transcription in response to telomere damage may be a part of telomere signaling affecting various cellular processes [22]. An important question in *Drosophila* telomere biology is whether the level of *HeT-A* RNA could play a role in cellular response to the state of telomeres. To address this question, it is critical to examine the *HeT-A* RNA and *HeT-A* Gag biogenesis in normal development and with telomere dysfunctions.

Previously, we have shown that the mechanisms of chromosome end protection are closely related to the silencing of telomeric repeats: upon depletion of the piRNA pathway components and other factors suppressing *HeT-A* expression, abundant long telomeric RNAs accumulate in the germline, then transported to the oocyte and form aggregates at the mitotic spindles during the syncytial stage of embryogenesis [20]. This phenotype is accompanied by severe mitotic defects, chromosome missegregation and leads to early embryonic lethality [20, 21]. However, the functional link between accumulation of telomeric transcripts upon telomere dysfunction and developmental defects remained unclear. To advance the understanding of the interplay between dysfunctional telomeres and various cellular pathways, we explored the localization and functional interactions of abundant *HeT-A* RNA and Gag protein in the germline and early development upon piRNA pathway disruption.

RNA-binding proteins are involved in RNA metabolism at different stages of its life cycle, from transcription to degradation. Transcripts and proteins encoded by *HeT-A* and *TART* were detected at different stages of germline development in wild type *Drosophila* strains as well as upon depletion of telomere silencing components [20, 23, 24]. Despite being crucial for telomere targeting and lengthening, the biogenesis of *Drosophila* telomeric RNP in the germline and in early development is still poorly understood since most studies are focused on either *HeT-A* and *TART* RNA or their protein products separately.

Due to robust piRNA-mediated silencing in the germline, telomeric retrotransposon products are present at very low levels in wild type ovaries. Even in the *Gaiano* strain which is characterized by increased *HeT-A* and *TART* copy numbers [25], *HeT-A* Gag protein is barely detected in germ cells [23], which can be explained by enhanced production of *HeT-A* piRNAs in *Gaiano* ovaries [26]. Valuable data on *Drosophila* telomeric RNP were recently obtained from studies of somatic tissues. *HeT-A* spherical particles consisting of *HeT-A* Gag and *HeT-A* RNA were discovered in proliferating neuroblasts where they associate with telomeres undergoing DNA replication and are considered a prerequisite for somatic *HeT-A* transposition [27].

Here, we show that upon piRNA loss overexpressed *HeT-A* transcripts interact with the RNA-binding protein Gag-*HeT-A*, which they encode, and that they form RNP particles during most of their life cycle in oogenesis and early embryogenesis. In germarium, *HeT-A* RNP form spherical structures in the nuclei of dividing cystoblasts and seem to be the intermediates of telomere elongation in the germline. It has been shown that interaction of *HeT-A* RNPs with the main carrier protein Egalitarian (Egl) [28] is required for their transport into the oocyte. Here, we describe for the first time the life cycle of maternal *HeT-A* RNP in early development. Retention of Egl at *HeT-A* RNP causes its ectopic localization at syncytial stage of embryogenesis which likely impairs embryonic development in the piRNA pathway mutants.

## Materials and methods

### *Drosophila* strains and transgenic constructs

Full-length *HeT-A* element encoding Gag protein tagged with HA (hemagglutinin) and FLAG epitopes was cloned into pUASp-attB as described previously [29]. To generate pUAST-attB-HeTA-HA-FLAG_ms2, 8 MS2 hairpins were introduced in the end of *HeT-A* 3`UTR. The construct pUAST-attB-HeTA-HA-FLAG-mut_ms2 was created on the basis of pUAST-attB-HeTA-HA-FLAG-ms2 by cloning of the mutated *HeT-A* hairpin (S6 Fig). Constructs were integrated in the attP docking site on chromosome 3 (strain #24862, BDSC). GLKD (Germline Knockdown) flies were F1 progeny of the genetic cross of a strain bearing a construct with short hairpin (sh) RNA (*spnE*_sh, #103913, VDRC; *piwi*_sh, #101658, VDRC; *egl*-sh, #21779, VDRC) and a driver strain #25751 (*P{UAS-Dcr-2*.*D}*. *v1*, *w*^*1118*^, *P{GAL4-nos*.*NGT}40*, Bloomington Stock Center). The transgenic strain expressing fused protein Egl-GFP was kindly provided by S. Bullock [28]. The *Gaiano III (GIII)* is a strain carrying a third chromosome with *Tel* locus derived from natural *Gaiano* strain characterized by extremely long telomeres [25].

### RNA immunoprecipitation (RIP)

RIP was performed according to [30] with modifications. Briefly, ovaries from 3-day old females or 0-2-hour old embryos were homogenized in the Dounce homogenizer (Sigma) in 5 volumes (V) of cold lysis buffer (10 mM HEPES, pH 7.0, 100 mM KCl, 5 mM MgCl_2_, 0.5% NP-40, Complete mini protease inhibitor cocktail (Roche), 20 mM NaF, 0.2 mM NaVO_4_, 1 U/μl RiboLock RNase Inhibitor (ThermoScientific)). For RIP with anti-HA beads, lysis buffer was supplemented with 1% Triton X-100, 0.1% SDS and 10% glycerol. The extracts were cleared by centrifugation at 16,000 g at 4° C for 10 min and diluted with 9 volumes of NT2 buffer (50 mM Tris–HCl, pH 7.4, 150 mM NaCl, 1 mM MgCl_2_, 0.05% NP-40, 0,2 mM NaVO_4_, 20 mM NaF, RiboLock 1U/μl). The lysates were incubated with anti-HA magnetic beads (Pierce) or with antibody-coated (5 μg of the primary antibodies per sample) Dynabeads Protein A (Invitrogen) for 2 h at 4°C on a rotator. After 3 washes in NT2 buffer, 1/10 of beads were saved for Western blot analysis. RNA was isolated from the remaining beads using Trizol reagent (Life Technologies). Reverse transcription was performed with a random hexanucleotide primer and SuperScriptIII reverse transcriptase (Life Technologies) according to the manufacturer’s instructions. qPCR was performed on a LightCycler96 (Roche). Primers are listed in S1 Table. Antibodies used for RIP: rabbit anti-EGL [31]; rabbit anti-GFP (Abcam), rabbit anti-HA (Cell signaling technology), normal rabbit IgG (Santa Cruz).

### RNA FISH and immunostaining

RNA FISH combined with immunostaining was carried out according to the previously described procedure [20] with modifications. After fixation, ovaries or dechorionized embryos were incubated in PBS containing 50 ug/ml proteinase K for 6 or 2 min, respectively. Proteinase K treatment was omitted in case of further staining for cytoplasmic proteins in the ovaries. Digoxigenin (DIG)-labeled antisense *HeT-A* riboprobe containing a fragment of the ORF (nucleotides 4330–4690 of GenBank sequence DMU06920) was used. The specificity of this probe was previously confirmed by Northern blotting and *in situ* hybridization with polytene chromosomes of salivary glands [19, 26]. To amplify hybridization signal, we incubated the sample with anti-DIG- fluorescein (FITC) antibodies (Roche), followed by incubation with anti-FITC Alexa Fluor 488 antibodies (Life Technologies). Blocking solution was supplemented with 3% BSA before immunostaining. Samples were mounted in Vectashield Antifade Mounting Medium with DAPI (Vector Laboratories). Images were captured using Zeiss LSM 510 Meta or Olympus FV10i confocal microscopes and analyzed using ImageJ and Adobe Photoshop.

### Co-immunoprecipitation (co-IP)

To prepare embryonic extracts, 0-2-hour old embryos were dechorionated, frozen and stored at -80°C. Ovaries (400 pairs) or embryos (the volume of dechorionized embryos ~70 μl) were homogenized in a Dounce homogenizer in 9 volumes of cold IP Buffer (20 mM HEPES pH 7.0, 150 mM NaCl, 2,5 mM MgCl_2_, 0.1% Triton X-100, Complete Mini protease inhibitor cocktail (Roche), 0,2 mM NaVO_4_, 20 mM NaF, RiboLock 1U/μl (ThermoScientific)). The extracts were cleared by centrifugation at 16,000 g at 4°C for 10 min. Supernatants were incubated with anti-HA magnetic beads (20 μl, Pierce) for 30 min at room temperature on a rotator. After 3 washes in IP buffer the bound proteins were eluted from the beads by boiling in 100 μl of Laemmli protein loading buffer (31,25 mM Tris-HCl, pH 6.8, 12,5% glycerol, 1% SDS, 0.005% Bromophenol Blue, 2,5% β-mercaptoethanol) for 5 min. Beads were pelleted and the supernatant was saved for Western blot analysis. Samples were resolved on 8% SDS-PAGE gel and transferred onto Immobilon-P PVDF membrane (Millipore). Blots were developed using the Immun-Star AP detection system (Bio-Rad Laboratories), in accordance with the manufacturer’s recommendations.

### Antibodies

The following primary antibodies were used: rabbit anti-Egl [31] (kindly provided by R. Lehmann); mouse anti-Dhc (Developmental Studies Hybridoma Bank; donor J. Scholey); mouse anti-BicD (Developmental Studies Hybridoma Bank, donor R. Steward); rat anti-Vasa (Developmental Studies Hybridoma Bank; donors A.C. Spradling/D/Williams); rabbit anti-gamma-tubulin (Sigma); mouse anti-GFP (Abcam); mouse anti-HA (Cell signaling technology); rabbit anti-HA (Cell signaling technology); rabbit anti-HOAP, guinea pig anti-HipHop and anti-Gag HeT-A (kindly provided by Y. Rong); rabbit anti-Gag HeT-A (kindly provided by E. Casacuberta (23) Alexa fluor conjugated secondary antibodies (Life Technologies) were diluted 1:500.

## Results

### *HeT-A* RNA and *HeT-A* Gag are co-localized in ovaries and form *HeT-A* RNPs at different stages of oogenesis

RNA biogenesis is orchestrated by RNA-binding proteins that determine the localization, lifetime and the specificity of transcript interactions. Therefore, in order to understand the role of telomeric coding RNAs, it is first necessary to identify their protein partners. Direct recognition of the retrotransposon transcript by the protein it encodes was discovered for human retrotransposon *LINE-1* (*L1*) and is referred to as cis-preference [32, 33]. We suggested that the *Drosophila* telomeric protein Gag, encoded by *HeT-A*, interacts with *HeT-A* RNA forming RNP in the germline. Recombinant engineering of epitope tags and inducible tissue-specific expression of transgene containing full-length *HeT-A* have made detection and purification of *HeT-A* Gag complexes routine [29]. Due to the activity of the piRNA pathway and other telomere silencing mechanisms, expression of *HeT-A* is strongly repressed in the germline [13, 20]. Indeed, *HeT-A* Gag is not detectable by Western blotting in the ovaries of *GIII* and transgenic strains expressing HeT-A-HA in contrast to somatic tissues (S1 Fig). However, upon piRNA loss, caused by depletion of the RNA helicase Spindle-E (SpnE) [34], abundant *HeT-A* Gag accumulates in the ovaries (Part A in S1 Fig). To get an insight into the biogenesis and pathological significance of abundant telomeric RNAs and proteins generated upon telomere dysfunction, we characterized their localization during oogenesis and first hours of development after germline knockdown (GLKD) of *spnE*.

**Fig 1:**
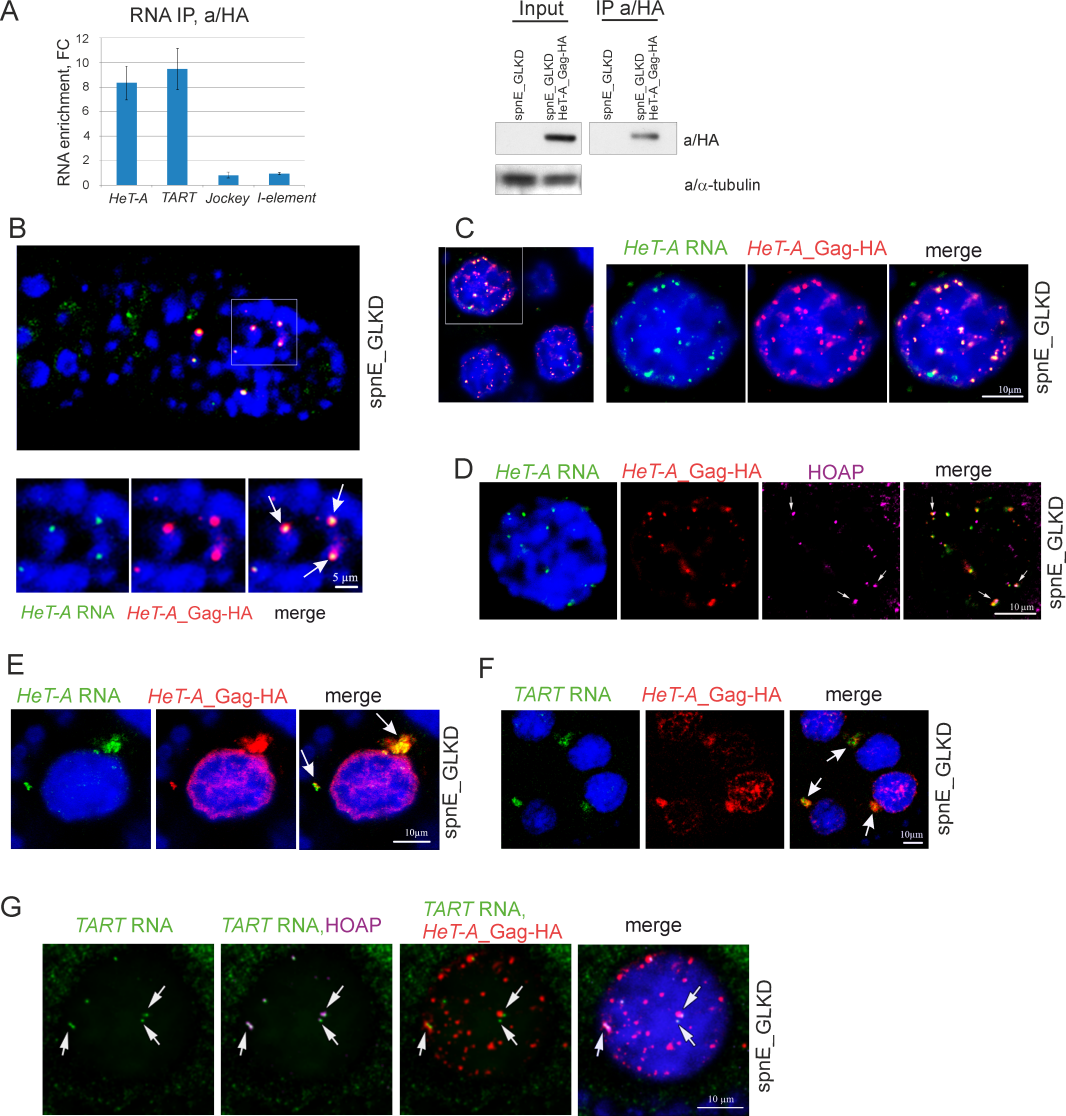
*HeT-A* RNA and *HeT-A* Gag protein form RNP in ovaries. **(A)** RT-qPCR analysis of RNA immunoprecipitated with anti-HA from ovary lysates of nosGal4; UAS-HeT-A-HA; UAS-spnE_sh flies and control nosGal4; UAS-spnE_sh flies. Fold changes (FC) for RNA enrichments in RNA IP from ovaries of HeT-A-HA transgenic strain versus control are shown. *rp49* was used for normalization. The error bars represent standard deviations (SD) of 2 biological replicas. Western blot analysis of immunoprecipitated *HeT-A* Gag-HA is shown to the right. The antibodies used for Western blotting are indicated to the right. (B-G) RNA FISH combined with immunostaining was performed on ovaries of nosGal4; UAS-HeT-A-HA; UAS-spnE_sh flies. (B) *HeT-A* RNA (green) colocalizes with *HeT-A* Gag-HA (red) in germarium. Enlarged region (white rectangle) is shown. *HeT-A* spheres (arrows) are observed in cystoblasts. (C) *HeT-A* RNA (green) colocalizes with *HeT-A* Gag-HA (red) in nurse cell nuclei. A fragment of a stage 7 egg chamber is shown to the left. An enlarged nucleus of a germline nurse cell (white rectangle) is shown. (D) *HeT-A* RNA FISH (green) combined with *HeT-A* Gag-HA (red) and HOAP (magenta) immunostaining in a nurse cell nucleus from a stage 7 egg chamber is shown. Telomeric localization of *HeT-A* RNP is shown by arrows. (E) *HeT-A* RNA (green) and *HeT-A* Gag-HA (red) form large aggregates (arrows) in the cytoplasm of nurse cells. A fragment of a stage 5 egg chamber is shown. For an entire egg chamber see Part A in S2 Fig. (F) *TART* transcripts (green) are co-localized with *HeT-A* Gag-HA (red) in the nurse cell cytoplasm (arrows). A fragment of a stage 7 egg chamber is shown. (G) *TART* RNA (green) colocalization with telomere-specific protein HOAP (magenta) is shown (arrows). *HeT-A* Gag-HA (red) partially colocalizes with *TART* RNA in nurse cell nuclei. Image of an individual nurse cell nucleus from a stage 6 egg chamber.

To determine whether *HeT-A* Gag associates with *HeT-A* RNA, a RIP experiment followed by RT-qPCR of co-precipitated RNA was performed. Ovarian lysates expressing *HeT-A* Gag-HA upon *spnE_*GLKD were immunoprecipitated with a/HA antibodies. Ovaries of *spnE*_GLKD lacking the UAS-HeT-A-HA transgene were used as controls (Fig 1A). *HeT-A* transcripts are significantly enriched in the *HeT-A* Gag-HA precipitate (Fig 1A). Interestingly, endogenous *TART* RNA was also present in this complex suggesting that *HeT-A* RNPs are multicomponent complexes.

Next, we analyzed the co-localization of *HeT-A* RNA and Gag protein at different stages of oogenesis upon *spnE*_GLKD using RNA fluorescence *in situ* hybridization (FISH) combined with immunostaining. In the germarium region 2A, *HeT-A* transcripts co-localize with Gag-positive particles forming spherical RNPs of 1–2 microns in size (Fig 1B) resembling those discovered in brain cells [27]. Numerous *HeT-A* spheres observed in the nuclei of dividing cystoblasts are presumably intermediates of the telomere elongation complex, in agreement with the high rate of telomeric attachments in *spnE* mutants [13]. We failed to detect spherical *HeT-A* RNPs in the female germ cells of *GIII* strain (Parts B and C in S1 Fig). Taking into account the extremely low rate of spontaneous terminal transpositions [35], assembly of *HeT-A* spheres appears to be a rare event under normal conditions.

During the later stages of oogenesis, *HeT-A* RNPs are detected in the nurse cells and oocyte upon piRNA loss. In the oocyte, *HeT-A* Gag and *HeT-A* RNA staining are evenly distributed and extensively overlapped (Part A in S2 Fig). Most of the nuclear *HeT-A* RNP in the nurse cells have a spherical shape (Fig 1C), however, only some of them co-localize with telomeres stained for telomere-specific proteins HOAP or HipHop [36, 37] (Fig 1D and Part B in S2 Fig). We observed similar patterns of *HeT-A* RNP localization both for endogenous and transgenic *HeT-A*, which argues against nonspecific effects caused by transgene overexpression (Part C in S2 Fig). *HeT-A* RNA FISH combined with HA-immunostaining on wild type *yw* ovaries served as a negative control (Part D in S2 Fig). In the nurse cell cytoplasm, some of the *HeT-A* RNP foci form large aggregates of irregular shape differed from nuclear spherical particles (Fig 1E). *HeT-A* Gag-HA staining was also revealed in the perinuclear ribonucleoprotein structure—the nuage. However, immunostaining of *HeT-A* Gag and a nuage component Vasa shows only partial colocalization of these proteins (Part A in S3 Fig). *HeT-A* Gag staining is more diffuse and found not only around the nucleus but also in the nucleus close to the envelope. Most likely, nuclear import of abundant *HeT-A* Gag is accompanied by its sequestration in the nuage.

In accordance with RIP, *TART* sense transcripts co-localize with aggregates of *HeT-A* RNP in the nurse cell cytoplasm (Fig 1F). In nurse cell nuclei, *TART* transcripts are revealed nearby telomeres but not in the *HeT-A* spheres (Fig 1G).

All these data taken together suggest that *HeT-A* RNA and *HeT-A* Gag form RNPs of different structures at different stages of oogenesis upon loss of piRNA silencing.

**Fig 2:**
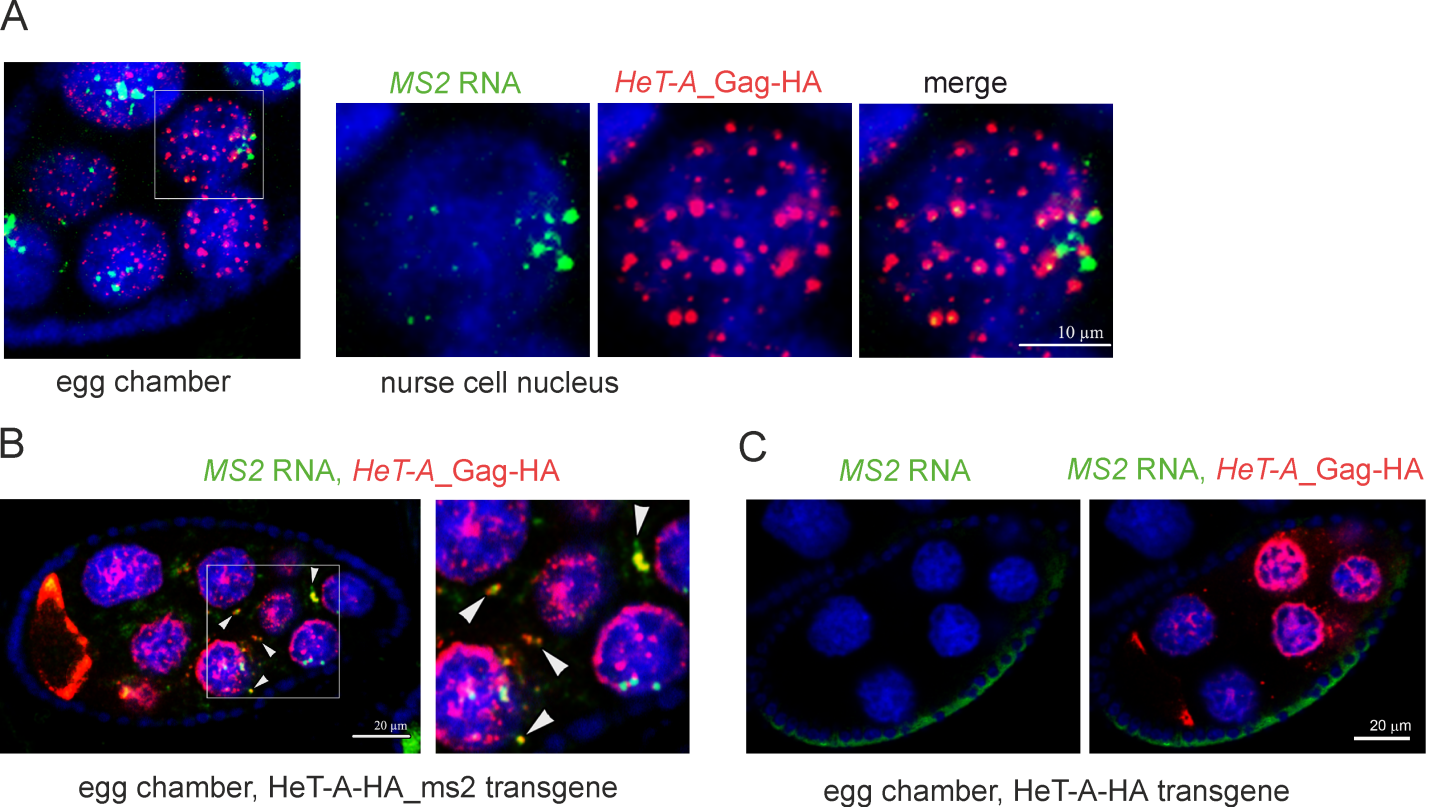
*HeT-A* Gag demonstrates cis-preferential activity to the template *HeT-A* mRNA. (A) *MS2* RNA FISH combined with anti-HA immunostaining was performed on ovaries of a transgenic strain expressing HeT-A-HA-ms2 upon *spnE*_GLKD. Transgenic *HeT-A-HA_ms2* RNA (green) colocalizes with *HeT-A* Gag-HA (red) in nurse cell nuclei. A stage 6 egg chamber and enlarged nurse cell nucleus (rectangle) are shown. Intensive *HeT-A* signals in the nurse cell nuclei most likely correspond to the actively transcribed *HeT-A* transgenes. (B) *MS2* RNA (green) and *HeT-A* Gag-HA (red) form large aggregates (arrowheads) in the cytoplasm of nurse cells of transgenic strain expressing HeT-A-HA-ms2 upon *spnE*_GLKD. A stage 7 egg chamber and its enlarged fragment (rectangle) is shown. (C) *MS2* RNA FISH (green) and HA immunostaining (red) on ovaries of transgenic strain expressing HeT-A-HA upon *spnE*_GLKD serves as a negative control and confirms specificity of the *MS2* probe.

In order to demonstrate direct interaction between the *HeT-A* Gag protein and its RNA template (cis-preference rule) we took advantage of another transgenic strain containing a *HeT-A* copy, tagged by the MS2 bacteriophage hairpins, and encoding for Gag-HA. Combined *MS2* RNA FISH and HA-immunostaining shows colocalization of transgenic RNA and protein in the nuclei and cytoplasm of germ cells (Fig 2). Distribution patterns of transgenic *HeT-A* RNPs are similar to that of endogenous *HeT-As*. However, due to the lower sensitivity of the *MS2* probe, we used *HeT-A* ORF probe in further experiments. To rule out artifacts due to nonspecific *MS2* RNA probe hybridization we performed control experiment. The *MS2* probe does not recognize nontagged *HeT-A-HA* RNA in transgenic ovaries expressing *HeT-A* Gag-HA upon *spnE*_GLKD (Fig 2C).

We conclude that *HeT-A* RNA and Gag form RNP according to the cis-preference rule.

### *HeT-A* RNPs interact with Egl in ovaries and form granules in the cytoplasm of nurse cells and oocyte

**Fig 3:**
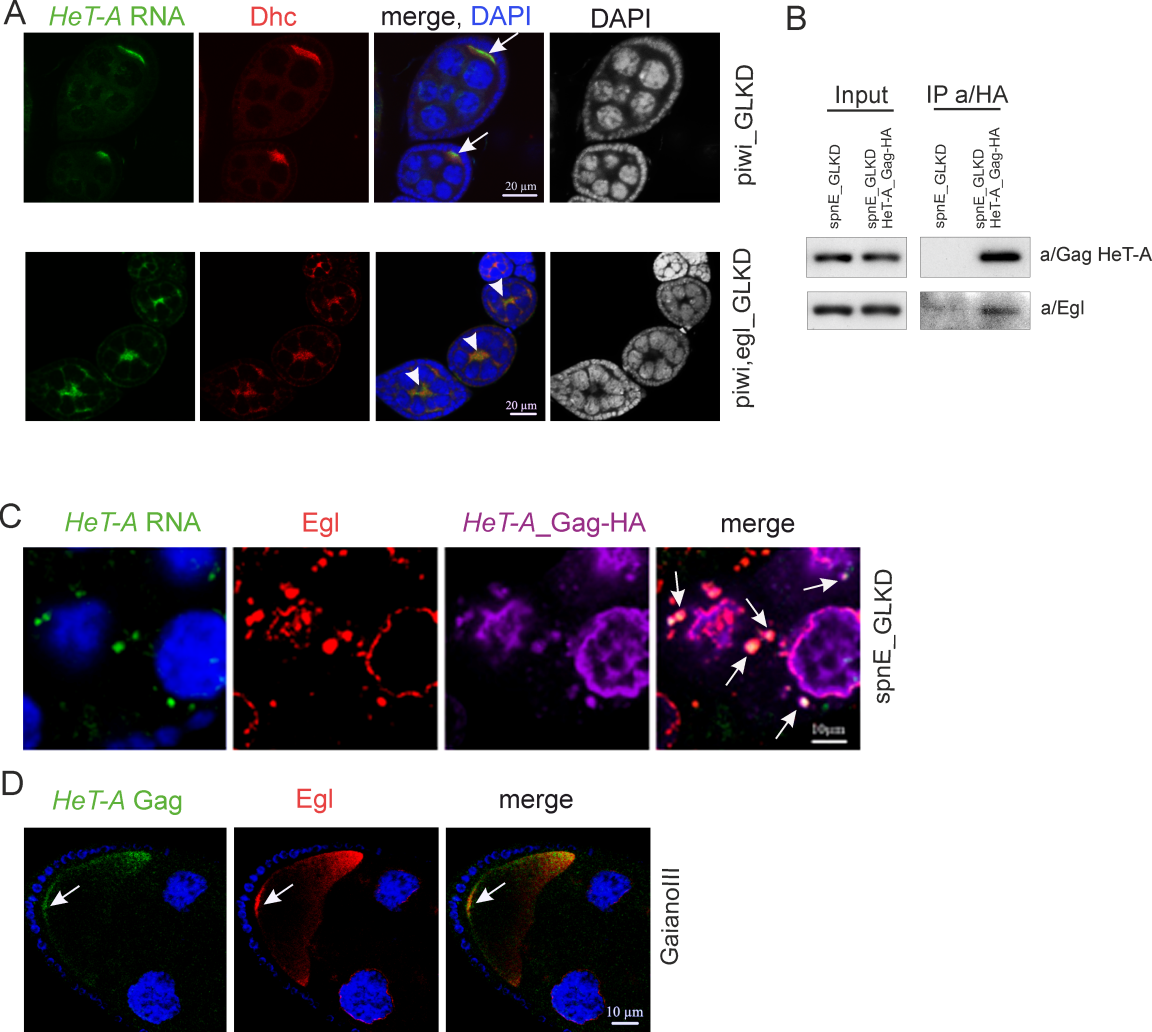
Egl participates in the transport of *HeT-A* RNPs in ovaries. (A) Egl germline knockdown results in mis-localization of *HeT-A* transcripts. Egg chambers at stage 4–5 of oogenesis from *piwi*_GLKD (top panel) and double *piwi-egl*_GLKD (bottom panel) are stained with a/Dynein heavy chain (Dhc) (red). *HeT-A* RNA (green) is localized in the oocyte upon *piwi*_GLKD (arrows), but is mis-localized upon *piwi*,*egl* double GLKD (arrowheads). Ectopic Dhc is observed at this stage according to published data [40]. DAPI is shown separately in a grey. (B) Co-IP of *HeT-A* Gag. Western blot analysis of proteins immunoprecipitated with anti-HA from ovaries of spnE-GLKD flies (control) and nosGal4; UAS-HeT-A-HA; UAS-spnE_sh. Anti-HA immunoprecipitates *HeT-A* Gag-HA and Egl proteins. The antibodies used for Western blotting are indicated to the right and the antibodies used for co-IP are indicated above the IP lanes. (C) *HeT-A* RNA (green), *HeT-A* Gag-HA (magenta) and Egl (red) form aggregates (arrows) in the cytoplasm of the nurse cells in ovaries of nosGal4; UAS-HeT-A-HA; UAS-spnE_sh flies. The fragment of a stage 9 egg chamber is shown. For an entire egg chamber see Part A in S5 Fig. (D) *HeT-A* Gag (red) and Egl (green) co-localize at the posterior pole of the oocyte (arrows) at stage 10 in ovaries of *GIII* strain. A fragment of a stage 10 egg chamber including oocyte is shown. DNA is stained with DAPI (blue).

Upon piRNA pathway disruption, *HeT-A* products accumulate in the oocyte from the early stages of oogenesis, suggesting that some mechanism provides their relocation from the nurse cells to oocyte [13, 24] (Part A in S2 Fig). Egl, a protein that interacts with the dynein motor complex, is a major carrier which provides the transportation and localization of maternal RNAs at different stages of oogenesis [28, 31, 38]. Egl is essential for oocyte determination since in Egl mutants all 16 germ cells of the egg chamber become nurse cells [39, 40]. We therefore suggested that Egl might be involved in *HeT-A* RNA localization. In ovaries with double *piwi*, *egl* GLKD, *HeT-A* specific localization is not observed (Fig 3A). Both *HeT-A* RNA and dynein accumulated in the cytoplasm of nurse cells. *HeT-A* delocalization is likely caused by the severe disorganization of microtubules caused by Egl depletion [39, 40]. Based on this observation, we focused on examining the role of Egl in *HeT-A* RNP transport.

To determine whether Egl directly associates with *HeT-A* RNAs, we performed RIP followed by RT-qPCR. RIP was performed on ovaries with a *HeT-A* overexpression or wild type background. Lysates of *spnE* GLKD ovaries expressing *HeT-A* Gag-HA were immunoprecipitated with a/Egl or normal rabbit serum. Alternatively, Egl-GFP was immunoprecipitated using rabbit a/GFP or normal rabbit IgG from ovary lysate of Egl-GFP strain at a wild type background. As expected, *nanos (nos)*, *oskar* (*osk)* and *gurken (grk)* mRNAs are associated with Egl-BicD complex in wild type ovaries (S4 Fig) [38]. It is worth noting, that association of *nos*, *osk* and *grk* transcripts with Egl is not affected by *spnE*_GLKD (S4 Fig). However, *HeT-A* RNA enrichment was insignificant in both cases, and *HeT-A* Gag is undetectable in Egl-BicD complex (S4 Fig). Of note, the majority of *HeT-A* Gag is present in an insoluble pellet after tissue lysis indicating its strong tendency to aggregate.

We suggested that if Egl does not associate directly with *HeT-A* RNA, it probably interacts with *HeT-A* Gag, and that this interaction is sensitive to the stringent purification conditions used in RIP (see Materials and methods). Co-IP experiments on *spnE*_GLKD ovaries using an HA antibody show that Egl is co-purified with *HeT-A* Gag-HA (Fig 3B) confirming that Egl indeed associates with transgenic *HeT-A* Gag. *HeT-A* RNA FISH combined with immunostaining confirmed the co-localization of Egl and *HeT-A* RNPs in the cytoplasm of the nurse cells and oocyte (Fig 3C and Part A in S5 Fig). In the nurse cells, Egl and *HeT-A* RNP form large aggregates of irregular shape in the cytoplasm and are partially colocalized in the nuage (Figs 3C and S3). Immunostaining of Egl and endogenous *HeT-A* Gag in ovaries of *spnE*_GLKD lacking the UAS-HeT-A-HA transgene revealed numerous Egl granules co-localized with *HeT-A* Gag indicating that their formation is not a side effect of transgenic Gag-HA aggregation (Part B in S5 Fig). In agreement with this observation, Co-IP experiments on *spnE*_GLKD ovaries lacking the *HeT-A* transgene show that Egl is co-purified with endogenous *HeT-A* Gag (Part C in S5 Fig).

We proposed that in the wild type ovaries, *HeT-A* RNP also interact with Egl and therefore performed Egl and *HeT-A* Gag immunostaining combined with *HeT-A* RNA FISH on ovaries of the *GIII* strain, which is characterized by an increased *HeT-A* copy number [25]. Egl was previously shown to be localized at the posterior pole of oocytes at stages 9–10 of oogenesis [31, 38]. Despite the low level of *HeT-A* Gag in *GIII* ovaries, we detected its enrichment at the posterior pole and co-localization with Egl (Fig 3D). This result confirms Egl association with endogenous *HeT-A* Gag in wild type ovaries.

Taken together, our data indicate that Egl carrier function is required for translocation and localization of *HeT-A* RNP in the oocyte upon *HeT-A* overexpression caused by piRNA loss. The experiment using the *GIII* strain indicates that probably the same mechanism operates in a wild type background.

### Association of abundant *HeT-A* RNP with Egl causes ectopic accumulation of Egl near to centrosomes in early embryos upon piRNA loss

**Fig 4:**
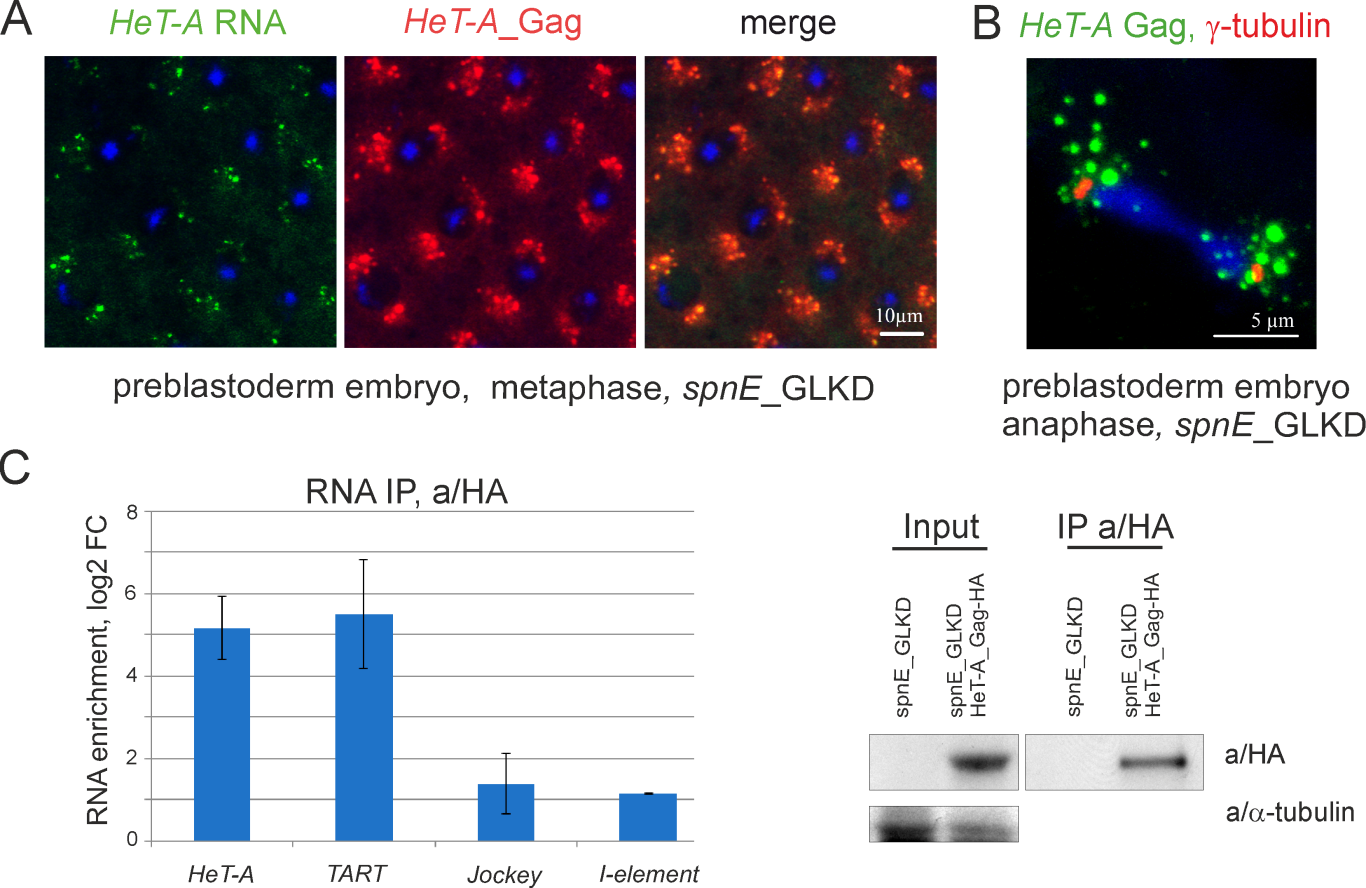
*HeT-A* RNA and *HeT-A* Gag form RNPs in early embryos. (A) Endogenous *HeT-A* RNA FISH (green) and anti-*HeT-A* Gag immunostaining (red) was performed on 0-2-hour old embryos after *spnE*_GLKD. Syncytial metaphase stage is shown. (B) *HeT-A* Gag (green) and gamma-tubulin (red) immunostaining was performed on 0-2-hour old embryos upon *spnE*_GLKD. Syncytial anaphase is shown. (C) RT-qPCR analysis of RNA precipitated with anti-HA from lysates of 0-2-hour old embryos laid by nosGal4; UAS-HeT-A-HA; UAS-spnE_sh flies relative to control nosGal4; UAS-spnE_sh embryos. log2 of fold changes (log2_FC) for RNA enrichments in RNA IP from embryos of HeT-A-HA transgenic strain versus control are shown. *rp49* was used for normalization. The error bars represent SD of 2 biological replicas. Western blot analysis of immunoprecipitated *HeT-A* Gag-HA is shown to the right. The antibodies used for Western blotting are indicated to the right.

Previously, we reported, that maternal *HeT-A* transcripts and transgenic *HeT-A* Gag-HA form aggregates around centrosomes during blastoderm formation in *spnE*_GLKD background [20, 29]. Specific localization of *HeT-A* RNA is dependent on microtubules since disruption of microtubules caused delocalization of *HeT-A* transcripts [29]. Similarly, endogenous *HeT-A* RNA and *HeT-A* Gag form granules and accumulate around centrosomes in 0-2-hour old embryos collected from *spnE*_GLKD females (Fig 4A and 4B). At this stage, *HeT-A* transcripts do not co-localize with the telomeres of mitotic chromosomes suggesting a non-telomeric role for *HeT-A* RNA.

First, we addressed a question whether *HeT-A* RNA and *HeT-A* Gag form RNP in early embryos. A RIP experiment was performed on lysates of 0-2-hour old embryos of a transgenic strain expressing UAS-HeT-A-HA in the germline upon *spnE_*GLKD. As a control, embryo lysates from non-transgenic *spnE*_GLKD flies were used (Fig 4C). *HeT-A* transcripts are significantly enriched in *HeT-A* Gag-HA precipitate from 0-2-hour embryos. Similar to RIP data on ovaries, *TART* RNA is also present in *HeT-A* RNPs of early embryos.

**Fig 5:**
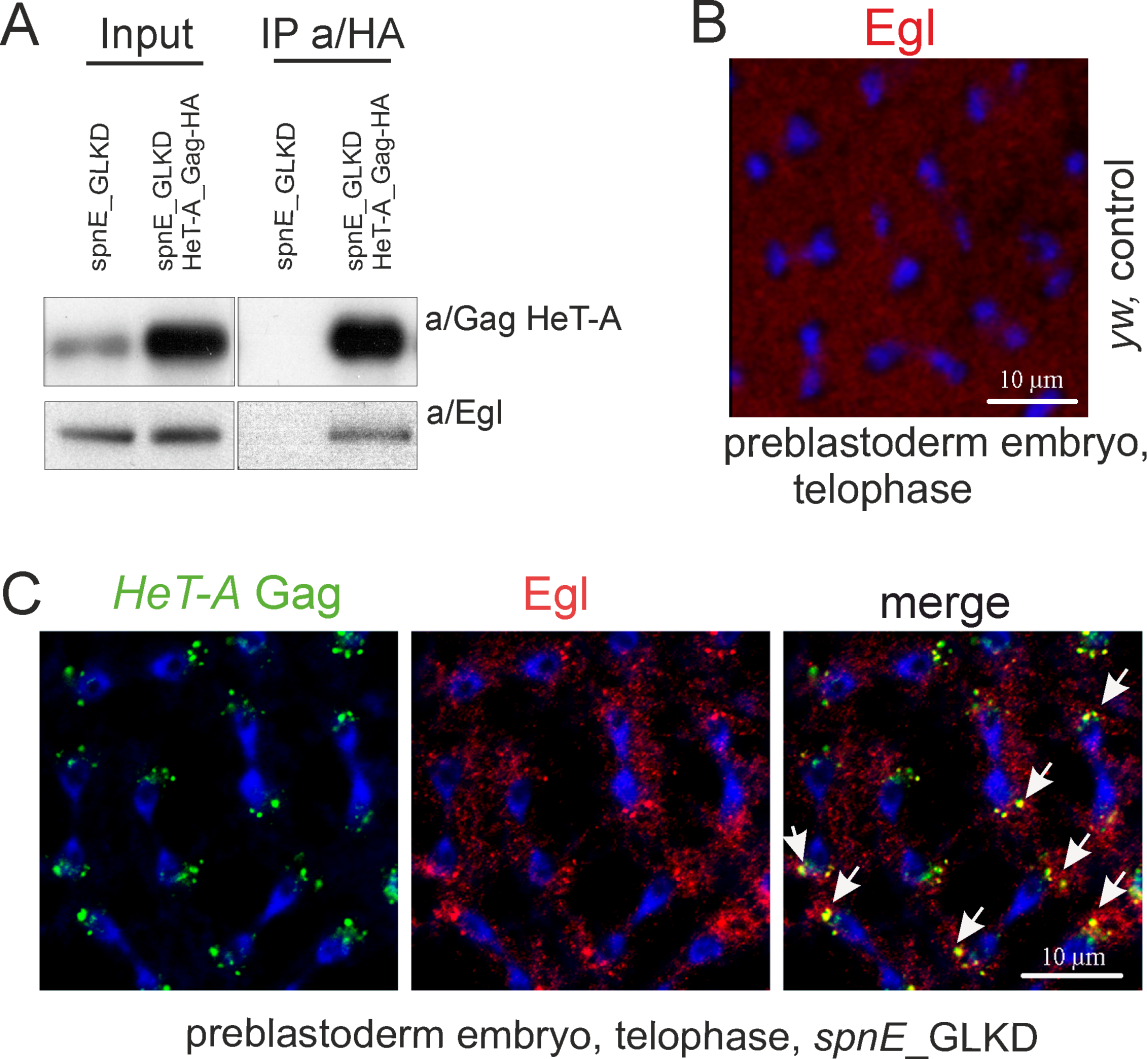
*HeT-A* RNPs associate with Egl in early embryos. (A) Western blot analysis of proteins immunoprecipitated with anti-HA from 0-2-hour old embryos laid by *spnE*_GLKD flies with and lacking the UAS-HeT-A-HA transgene. Anti-HA immunoprecipitates *HeT-A* Gag-HA and Egl proteins. The antibodies used for Western blotting are indicated to the right and the antibodies used for co-IP are indicated above the IP lanes. (B) 0-2-hour old embryos of *y*^*1*^ *w*^*67c23*^ strain were immunostained with anti-Egl (red). Telophase stage of syncytial preblastoderm embryo is shown. (C) Immunostaining of 0-2-hour old embryos collected from *spnE*_GLKD flies was performed with anti/Gag *HeT-A* (green) and anti/Egl (red) antibodies. DNA is stained with DAPI (blue). Telophase is shown. Endogenous *HeT-A* Gag colocalizes with Egl around spindle poles (arrows).

Next, we asked if Egl associates with *HeT-A* Gag during early embryogenesis. Co-IP using the lysate of 0-2-hour old embryos from *spnE*_GLKD flies expressing *HeT-A* Gag-HA shows that Egl co-purifies with transgenic *HeT-A* Gag-HA (Fig 5A). Immunostaining reveals co-localization of endogenous *HeT-A* Gag and Egl near the spindle poles in 0-2-hour old embryos upon *spnE*_GLKD while in wild type, Egl is evenly distributed in the syncytial embryo interior (Fig 5B and 5C). These data suggest that interaction with *HeT-A* Gag may cause Egl retention and its ectopic localization in early embryos with *spnE*_GLKD background.

It is known that mRNA stem-loop elements are important for recognition by protein carriers [41–43]. We suggested that the hairpin structure detected in the *HeT-A* 3'UTR (untranslated region) is presumably involved in the *HeT-A* RNA localization, and therefore, generated а transgenic strain, encoding *HeT-A* transcripts with impaired hairpin structure (S6 Fig). The mutated *HeT-A* construct contains sequences of bacteriophage MS2 hairpins which allowed us to specifically detect transgenic transcripts using an *MS2* probe. Transgenic strains containing HeTA-HA-mut_ms2 construct were used to decipher the role of the identified structural motif in the transport and localization of *HeT-A* mRNA. In *spnE* GLKD ovaries, *HeT-A-HA-mut_ms2* transcripts colocalize with *HeT-A* Gag-HA and Egl and accumulate in the oocyte just like endogenous *HeT-A* RNA (Part C in S6 Fig). In early embryos, *HeT-A-HA-mut_ms2* RNAs localize near to the centrosomes stained with gamma-tubulin (Part D in S6 Fig). Similar to endogenous *HeT-A* Gag, Gag-HA encoded by *HeT-A-HA-mut_ms2* transgenic RNA co-localizes with Egl close to spindle poles (Part E in S6 Fig.). These data show that this particular hairpin structure is dispensable for Egl-mediated transportation and localization of *HeT-A* RNA in ovaries and embryos.

## Discussion

In this study, we describe the life cycle of maternal telomeric RNPs in *Drosophila*. To study the localization of telomeric RNA in oogenesis and early development, we focused on the major telomere repeat *HeT-A* which is overexpressed upon piRNA pathway disruption. It was shown that in the ovaries and embryos *HeT-A* transcripts interact with the RNA-binding protein Gag-*HeT-A* that they encode forming RNP complexes. Using tagged transgenic *HeT-A*, we confirmed that this interaction occurs according to the cis-preference rule discovered previously for the human *L1* retrotransposon [32, 33].

*HeT-A* RNPs form structures with different characteristics during oogenesis upon piRNA loss. In the early stages of oogenesis, during cystoblast cell divisions, nuclear *HeT-A* RNPs form spherical particles which are seemingly participate in telomere elongation. *HeT-A* spheres were revealed in germarium germ cells with *spnE* depletion, but not in wild type ovaries. This fact is in agreement with extremely low levels of *HeT-A* expression and therefore, low transposition rate to chromosome termini in wild type flies [13, 35]. Accumulation of *HeT-A* spheres in cystoblast nuclei is in accordance with the observation that identical telomeric attachments to the terminally truncated chromosomes have been identified in several descendants from the same female bearing a heterozygous *spnE* mutation, suggesting that terminal elongation occurs at premeiotic stages of oogenesis [13]. The germarium region where we have seen *HeT-A* spheres, and where expression of *HeT-A*-lacZ transgene was previously detected [19], corresponds to the so-called “Piwi-less pocket”, a small developmental window when transposons escape host control and can transpose [44]. Interestingly, telomeres appear to be elongated at the same stage suggesting that telomere length control and transposon regulation are coupled.

During later stages of oogenesis in *spnE* deficient ovaries, numerous spherical *HeT-A* RNPs are also detected in the nuclei of terminally differentiated nurse cells, with only a few of them associated with telomeres. We speculate that *HeT-A* RNPs could act here independently of telomere elongation and be involved in dysfunctional telomere signaling. Indeed, mammalian telomeric factors are able to localize outside telomeres, where they can regulate the transcription of various genes [22].

In the cytoplasm of nurse cells, *HeT-A* products form irregularly shaped aggregates which appear to be the transportation cargoes. Thus, only a portion of *HeT-A* RNPs generated in the nurse cells are translocated into nucleus while others are transported into the oocyte. It is noteworthy, that compositionally and functionally distinct *L1* RNPs were also revealed in human cells [45]. Taking into account the strong tendency of *HeT-A* Gag to aggregate, one may suggest that formation of regular spherical particles revealed in the nuclei is compartment-specific and requires additional factors.

*HeT-A* Gag co-IP in combination with RNA FISH and immunostaining suggests that the Egl-dynein carrier complex provides migration of cytoplasmic *HeT-A* RNPs in the germline. In piRNA pathway mutant, cytoplasmic aggregates consisting of dynein, Egl and BicD proteins, as well as transcripts of *bicoid*, *grk* and retrotransposon *I*-element were detected in both nurse cells and the oocyte [46]. We also show that cytoplasmic *HeT-A* RNPs co-localize with Egl. Seemingly, *HeT-A* RNPs can catalyze the aggregation of their interactors. If this is the case, interaction between Egl-BicD complex and *HeT-A* RNP particles can promote the formation of Egl-positive granules upon *HeT-A* overexpression caused by the piRNA loss.

Telomere targeting is an essential but not the only function of *HeT-*A RNPs. Maternal telomeric RNPs transported to the oocyte have a specific destination during early development. *HeT-A* RNPs accumulate near to the centrosomes in 0-2-hour old syncytial embryos suggesting some interplay between telomeric and mitotic machinery. Indeed, *HeT-A* overexpression in the germline is followed by the severe mitotic defects during early development that leads to the embryonic lethality [20]. Physical interaction between *HeT-A* RNPs and transportation pathway components may be in part responsible for the developmental defects that accompany *HeT-A* overexpression. Indeed, Egl and BicD associate with dynein motors and mediate transport of RNAs essential for the initial oocyte specification and subsequent embryonic patterning [31, 38, 39, 41]. Egl retention at centrosomes in complex with *HeT-A* RNP may lead to mis-localization of its typical cargoes, *nos* and *osk* mRNAs, observed in early embryos upon piRNA pathway disruption [47, 48]. Indeed, we show that upon *spnE*_GLKD *nos*, *osk* and *grk* transcripts are still loaded into Egl-BicD complex. However, Egl aggregation and its ectopic localization caused by interaction with abundant *HeT-A* Gag could affect the localization pattern of developmental mRNAs leading to embryonic axis defects observed upon piRNA loss [47, 49, 50]. We speculate that the abundant telomeric RNPs generated upon telomere dysfunction could cause developmental arrest of such embryos, thus protecting genome integrity.

The exact nature of the signal responsible for *HeT-A* RNP enrichment in the oocyte remains elusive. The stem-loop structure in the 3’ UTR of maternal mRNAs mediates transport of these RNAs from the nurse cells into the oocyte and their specific localization in early embryos [28]. Egl despite lacking a canonical RNA-binding domain is an RNA-binding protein capable of direct recognition of RNA localization signals within transcripts of *grk*, *hairy*, *K10*, and the *I*-element retrotransposon [28]. However, our data show weak if any *HeT-A* RNA binding to Egl. In addition, mutation of a hairpin in the 3`UTR of *HeT-A*, that is similar to Egl- bound hairpins of certain mRNAs, did not affect *HeT-A* localization. The interaction of *HeT-A* Gag with Egl-BicD-microtubule transport components was instead revealed which supports the idea that protein-protein interactions are essential for *HeT-A* RNP transportation by the Egl-BicD complex.

Further dissection of the biochemical composition of *HeT-A* RNPs during oogenesis and early development will increase understanding of the telomeric retrotransposition mechanism as well as non-telomeric functions of *HeT-A* products. Other telomeric retroelements represented by a few copies per genome, *TART* and *TAHRE*, encode reverse transcriptases which are likely critical for *HeT-A* retrotranspositions. It is known that transiently expressed *HeT-A* and *TART* Gag proteins are able to form telomere-associated structures in cultured cells; *TART* Gag moves to telomeres only when co-expressed with *HeT-A* Gag [51]. Moreover, co-localization of *HeT-A* Gag and *TART* Pol proteins was observed by immunostaining in neuroblast nuclei [23]. According to our data, *TART* transcripts along with *HeT-A* RNA are bound to *HeT-A* Gag supporting the idea that *HeT-A* RNP is a core of the multicomponent telomeric RNP complex. The role of proteins encoded by *TART* and *TAHRE* in telomere targeting and retrotransposition remains to be determined.

## Conclusions

We show that upon piRNA depletion transcripts of telomeric retrotransposon *HeT-A* interact with the *HeT-A* Gag protein, that they encode, according to the cis-preference rule, to form RNP particles during oogenesis and early embryogenesis. Our data suggest that *HeT-A* RNPs are multicomponent complexes which are present both in the nuclei and cytoplasm of germ cells, and in preblastoderm embryos. Spherical *HeT-A* RNPs found in the germ cells of germarium supposedly perform telomere elongation. Cytoplasmic *HeT-A* aggregates interact with carrier protein Egl which mediates the transportation of the *HeT-A* RNPs to the oocyte. *HeT-A* Gag-Egl complex is detected in early embryos near to centrosomes after *HeT-A* overexpression caused by piRNA pathway disruption. Interaction between *HeT-A* RNP and Egl carrier protein which is essential for embryonic axis specification likely impairs embryonic development in piRNA pathway mutants.

## Supporting information

S1 Fig *HeT-A* expression in ovaries is repressed by piRNAs. (A) Detection of *HeT-A* Gag in ovaries and carcasses (imago after ovary dissection) by Western blotting. Extracts were prepared from the *GIII* strain (first lane) and transgenic strains expressing UAS-HeT-A-HA in the germline on a wild type background (second lane) or upon *spnE*_GLKD (third lane). Antibodies are indicated to the left. In ovaries, *HeT-A* Gag is detected only upon piRNA pathway disruption (*spnE*_GLKD). (B, C) *HeT-A* RNA FISH (green) combined with endogenous *HeT-A* Gag (red) immunostaining on ovaries of *GIII* strain. *HeT-A* RNPs are not reveled in the germ cells but detected in somatic cells of germarium (B, arrows) in accordance to previously published observation (23). Intensive *HeT-A* staining observed in the nurse cell nuclei appear to be correspond to the transcribed telomeres (arrows) (C).
(TIF)

S2 Fig *HeT-A* RNPs in oogenesis. **(A)** *HeT-A* RNA (green) and *HeT-A* Gag-HA (red) form *HeT-A* RNPs (arrows) in the cytoplasm of nurse cells in the ovaries of nosGal4; UAS-HeT-A-HA; UAS-spnE_sh flies. Egg chamber at stage 7 of oogenesis is shown. (B) Colocalization of *HeT-A* RNA (green), *HeT-A* Gag-HA (red) and telomeric protein HipHop (magenta) is indicated by arrows. Nurse cell nucleus of a stage 6 is shown. (C) *HeT-A* RNA FISH (green) combined with endogenous *HeT-A* Gag (red) immunostaining on ovaries of non-transgenic *spnE*_GLKD strain. A fragment of a stage 7 egg chamber is shown. (D) *HeT-A* RNA FISH (green) combined with *HeT-A* Gag (red) immunostaining was performed on ovaries of *yw* wild type strain. An egg chamber at stage 7 of oogenesis is shown. DNA is stained with DAPI (blue).
(TIF)

S3 Fig *HeT-A* Gag is localized around nucleus and partially overlapped with Vasa. Immunostaining of a nuage component Vasa (red) and *HeT-A* Gag-HA (green) (A) or Egl (green) (B) is shown. Stage 6 egg chambers of transgenic strains expressing UAS-HeT-A-Gag-HA in the germline in wild type background (bottom panels) or upon *spnE*_GLKD (top panels). Arrows indicate nuage. Egl colocalizes with Vasa in nuage, while *HeT-A* Gag-HA staining is more diffuse and only partially overlapped with Vasa. Magnification is 63x.
(TIF)

S4 Fig Analysis of interaction between *HeT-A* RNA and Egl-BicD complex. (A) RT-qPCR analysis of RNA precipitated with anti-Egl relative to negative control (normal rabbit IgG) from ovary lysates of nosGal4; UAS-HeT-A-HA; UAS-spnE_sh flies. *rp49* was used for normalization. Western blot analysis of co-immunoprecipitated proteins is shown to the right. The antibodies used for Western blotting are indicated to the right. The antibodies used for co-IP are indicated above the IP lanes. Lane designation: input (total lysate), pellet (insoluble fraction), IP (precipitates). Anti-Egl immunoprecipitates both Egl and BicD proteins but not HeT-A Gag which is enriched in insoluble fraction. (B) RT-qPCR analysis of RNA precipitated with anti-GFP relative to negative control (normal rabbit IgG) from ovary lysates of w; tub-Egl-GFP flies. Western blot analysis of co-immunoprecipitated proteins is shown to the right; the indications are as in (A). Anti-GFP immunoprecipitates both Egl-GFP and Egl as well as BicD indicating that Egl-BicD is an oligomeric complex. For RIP panels, the error bars represent SEM of 2 biological replicas.
(TIF)

S5 Fig Egl and *HeT-A* RNP form granules in ovaries upon *spnE* knockdown. (A) *HeT-A* RNA (green), *HeT-A* Gag-HA (magenta) and Egl (red) form granules (arrows) in the cytoplasm of nurse cells in ovaries of nosGal4; UAS-HeT-A-HA; UAS-spnE_sh flies. Egg chamber at stage 7 of oogenesis is shown. (B) Egl (red) and endogenous *HeT-A* Gag (green) form granules (arrows) in ovaries of *spnE*_GLKD not carrying UAS-HeT-A-HA transgene. DNA is stained with DAPI (blue). (C) Co-IP of *HeT-A* Gag. Western blot analysis of proteins immunoprecipitated with anti-Gag *HeT-A* from ovaries of *spnE*_GLKD flies. Anti-Gag *HeT-A* immunoprecipitates Egl protein. The antibodies used for Western blotting are indicated to the right and the antibodies used for co-IP are indicated above the IP lanes.
(TIF)

S6 Fig The role of stem-loop element in the transport and localization of *HeT-A* mRNA. (A) A putative HLS1 (*HeT-A* localization signal 1) is revealed in the 3’UTR of *HeT-A* copies (start corresponds to 5798 position of canonical *HeT-A*, DM06920). Folding of HLS1 is shown. (B) Sequence of mutated *HeT-A* hairpin is shown. (C) Colocalization of *MS2* RNA (green), *HeT-A* Gag-HA (magenta) and Egl (red) in the cytoplasm of nurse cells (arrowheads) and in the oocyte (arrows) in ovaries of nosGal4; UAS-HeT-A-HA-ms2_mut flies upon *spnE*_GLKD. Two egg chambers at different stages of oogenesis are shown. (D) *MS2* RNA FISH (green) combined with immunostaining of gamma-tubulin (red) was performed on 0-2-hour old embryos of nosGal4; UAS-HeT-A-HA-ms2_mut flies upon *spnE*_GLKD. DNA is stained with DAPI (blue). Syncytial metaphase is shown. (E) *HeT-A* Gag-HA (red) and Egl (green) immunostaining were performed on 0-2-hour old embryos of nosGal4; UAS-HeT-A-HA-ms2_mut flies upon *spnE*_GLKD. DNA is stained with DAPI (blue).
(TIF)

S1 Table Primers used in the study (5’-to-3’). (XLSX)
