# Supplementary figures and images for: Subcellular localization and Egl-mediated transport of telomeric retrotransposon HeT-A ribonucleoprotein particles in the Drosophila germline and early embryogenesis
Source: PLoS One. 2018 Aug 29;13(8):e0201787. doi: 10.1371/journal.pone.0201787 (PMC6114517; doi:10.1371/journal.pone.0201787)

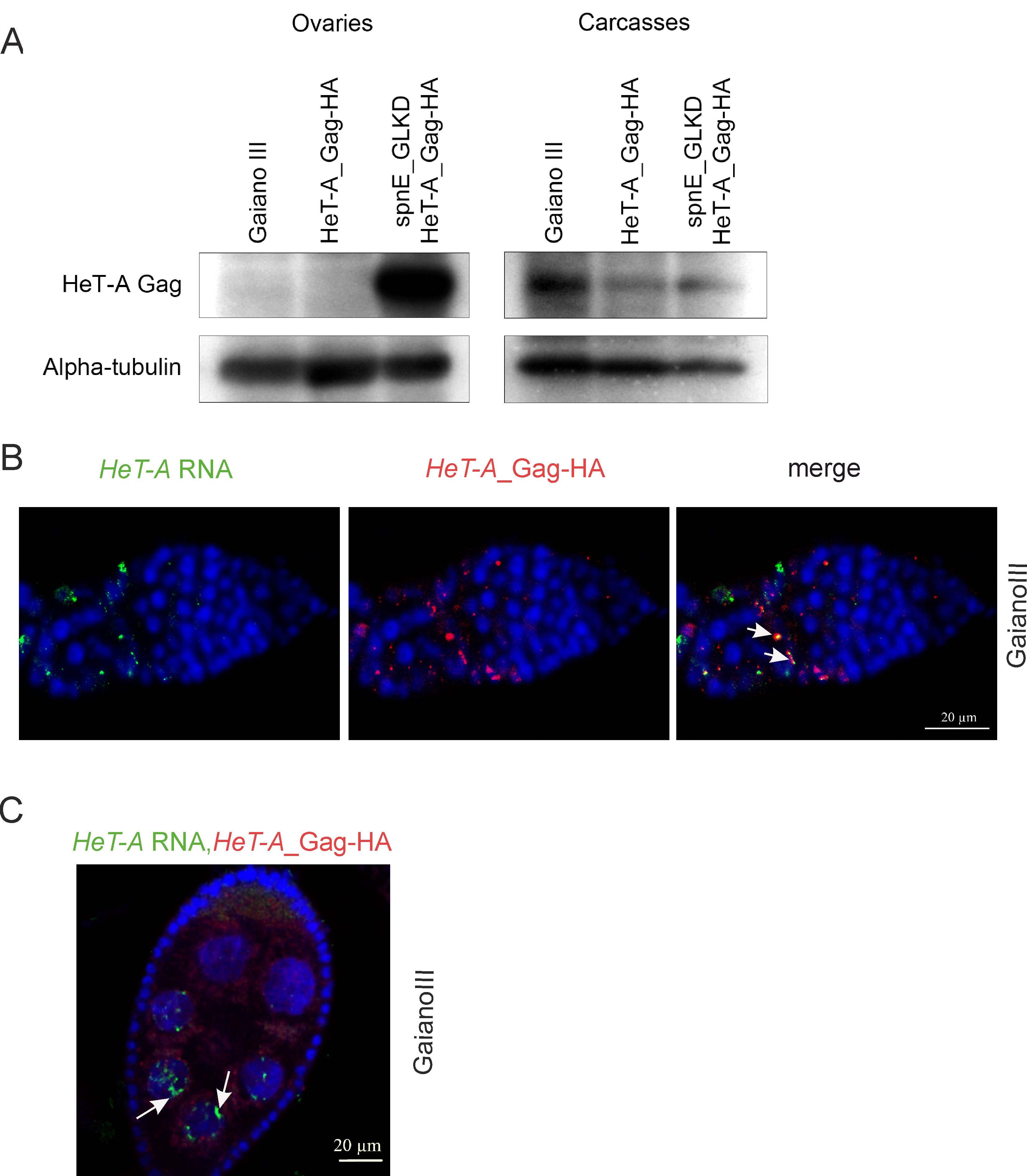

Supplement: S1 Fig — (A) Detection of HeT-A Gag in ovaries and carcasses (imago after ovary dissection) by Western blotting. Extracts were prepared from the GIII strain (first lane) and transgenic strains expressing UAS-HeT-A-HA in the germline on a wild type background (second lane) or upon spnE_GLKD (third lane). Antibodies are indicated to the left. In ovaries, HeT-A Gag is detected only upon piRNA pathway disruption (spnE_GLKD). (B, C) HeT-A RNA FISH (green) combined with endogenous HeT-A Gag (red) immunostaining on ovaries of GIII strain. HeT-A RNPs are not reveled in the germ cells but detected in somatic cells of germarium (B, arrows) in accordance to previously published observation (23). Intensive HeT-A staining observed in the nurse cell nuclei appear to be correspond to the transcribed telomeres (arrows) (C). (TIF) [file pone.0201787.s001.tif]

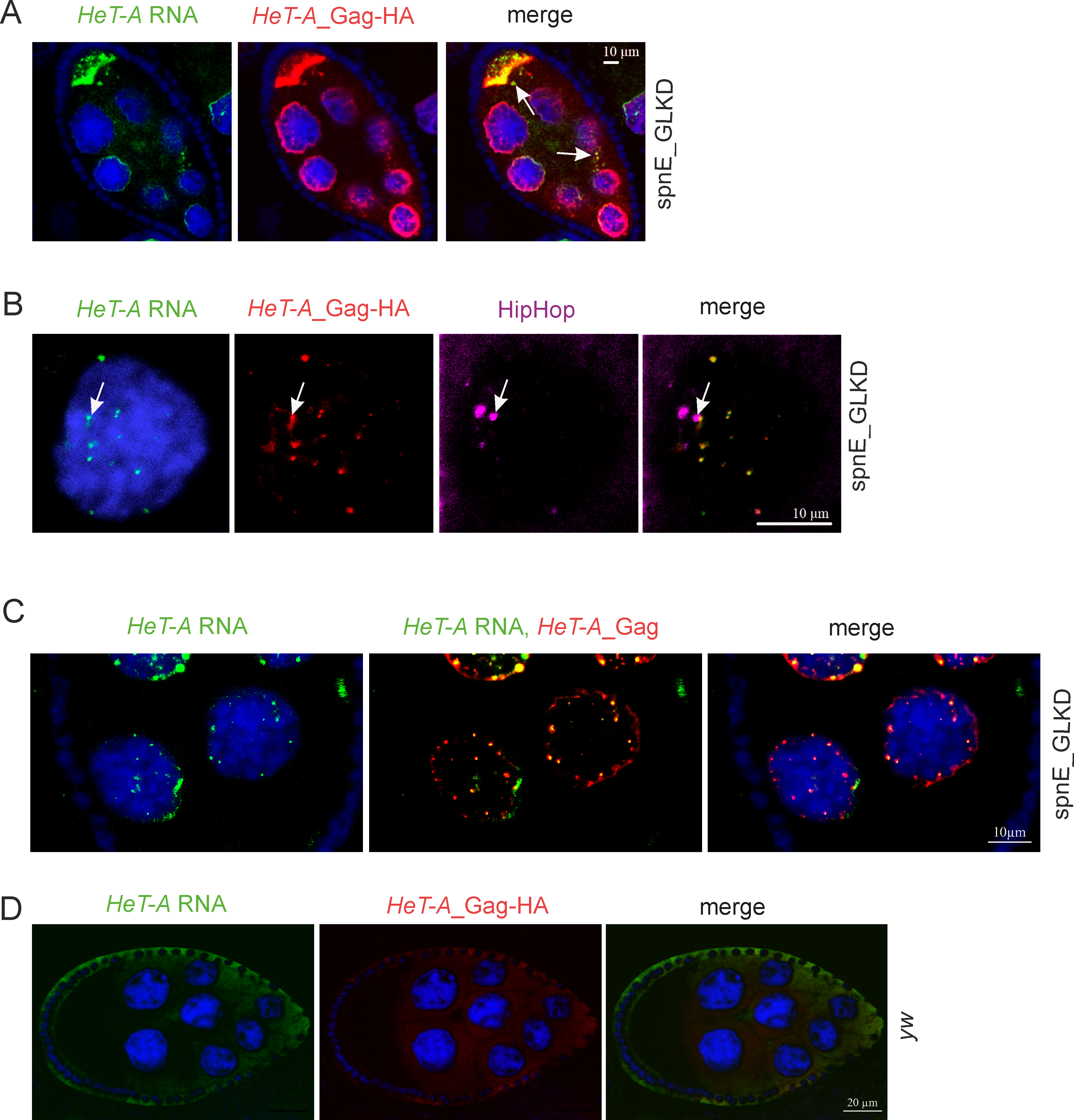

Supplement: S2 Fig — (A) HeT-A RNA (green) and HeT-A Gag-HA (red) form HeT-A RNPs (arrows) in the cytoplasm of nurse cells in the ovaries of nosGal4; UAS-HeT-A-HA; UAS-spnE_sh flies. Egg chamber at stage 7 of oogenesis is shown. (B) Colocalization of HeT-A RNA (green), HeT-A Gag-HA (red) and telomeric protein HipHop (magenta) is indicated by arrows. Nurse cell nucleus of a stage 6 is shown. (C) HeT-A RNA FISH (green) combined with endogenous HeT-A Gag (red) immunostaining on ovaries of non-transgenic spnE_GLKD strain. A fragment of a stage 7 egg chamber is shown. (D) HeT-A RNA FISH (green) combined with HeT-A Gag (red) immunostaining was performed on ovaries of yw wild type strain. An egg chamber at stage 7 of oogenesis is shown. DNA is stained with DAPI (blue). (TIF) [file pone.0201787.s002.tif]

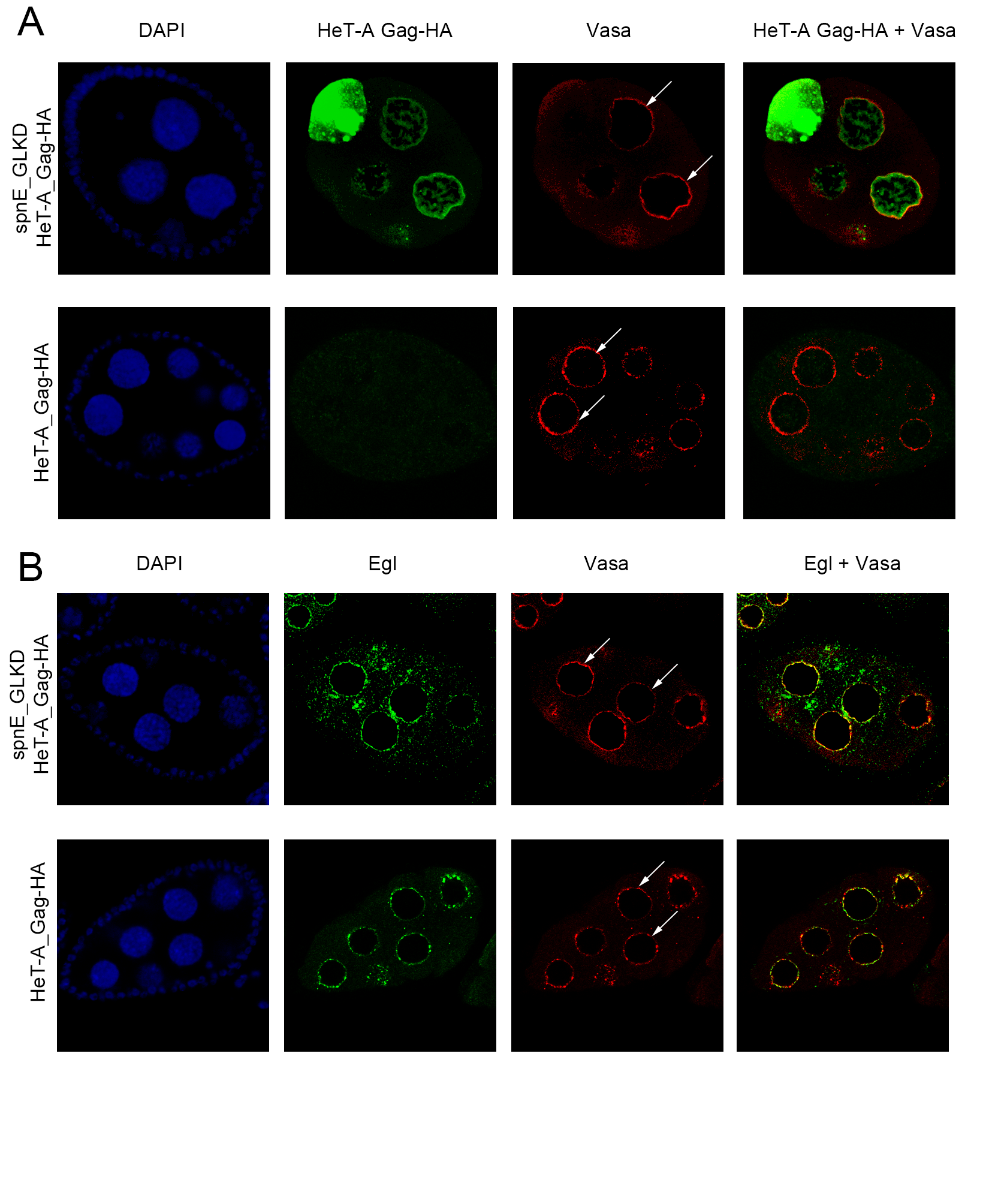

Supplement: S3 Fig — Immunostaining of a nuage component Vasa (red) and HeT-A Gag-HA (green) (A) or Egl (green) (B) is shown. Stage 6 egg chambers of transgenic strains expressing UAS-HeT-A-Gag-HA in the germline in wild type background (bottom panels) or upon spnE_GLKD (top panels). Arrows indicate nuage. Egl colocalizes with Vasa in nuage, while HeT-A Gag-HA staining is more diffuse and only partially overlapped with Vasa. Magnification is 63x. (TIF) [file pone.0201787.s003.tif]

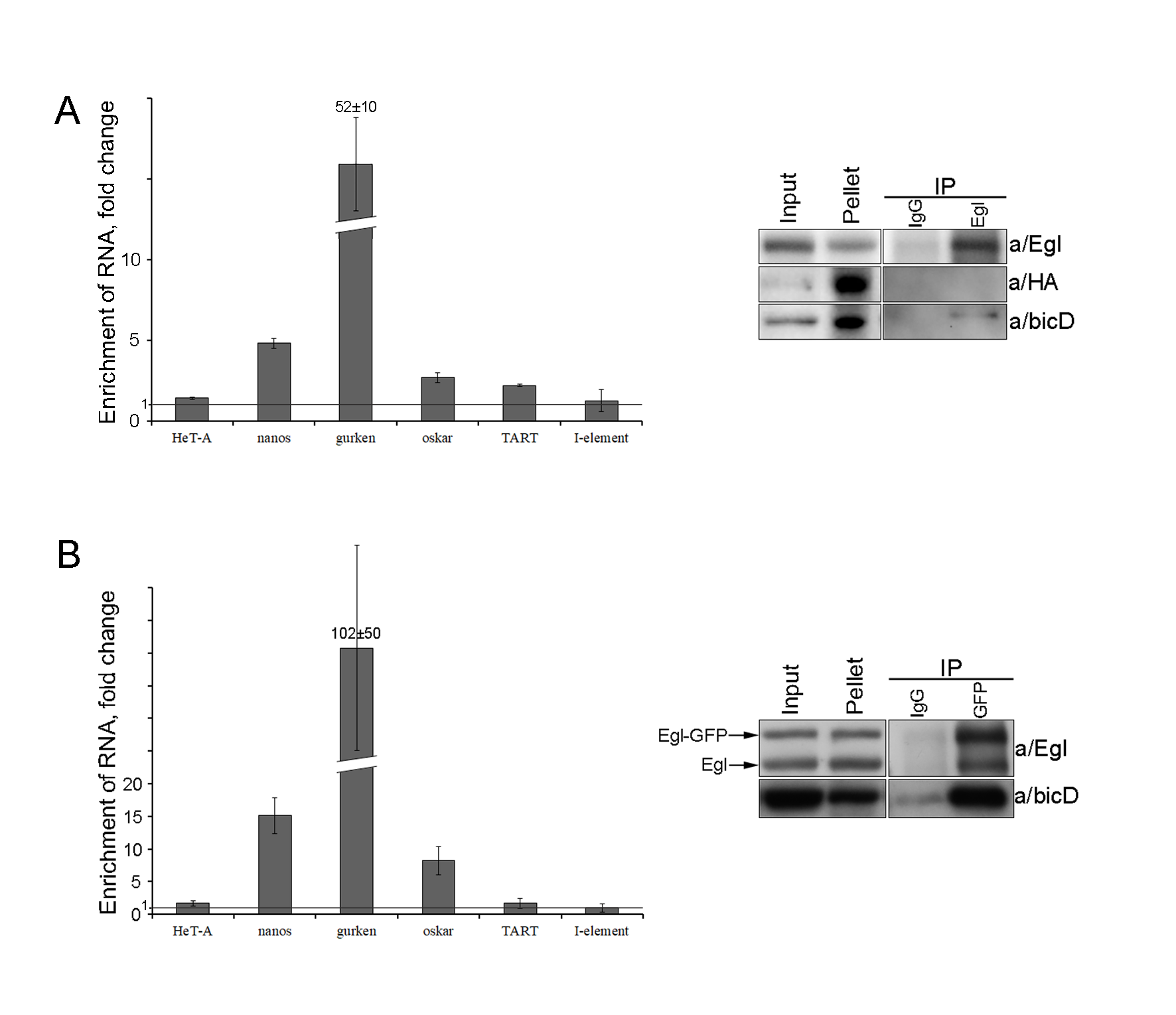

Supplement: S4 Fig — (A) RT-qPCR analysis of RNA precipitated with anti-Egl relative to negative control (normal rabbit IgG) from ovary lysates of nosGal4; UAS-HeT-A-HA; UAS-spnE_sh flies. rp49 was used for normalization. Western blot analysis of co-immunoprecipitated proteins is shown to the right. The antibodies used for Western blotting are indicated to the right. The antibodies used for co-IP are indicated above the IP lanes. Lane designation: input (total lysate), pellet (insoluble fraction), IP (precipitates). Anti-Egl immunoprecipitates both Egl and BicD proteins but not HeT-A Gag which is enriched in insoluble fraction. (B) RT-qPCR analysis of RNA precipitated with anti-GFP relative to negative control (normal rabbit IgG) from ovary lysates of w; tub-Egl-GFP flies. Western blot analysis of co-immunoprecipitated proteins is shown to the right; the indications are as in (A). Anti-GFP immunoprecipitates both Egl-GFP and Egl as well as BicD indicating that Egl-BicD is an oligomeric complex. For RIP panels, the error bars represent SEM of 2 biological replicas. (TIF) [file pone.0201787.s004.tif]

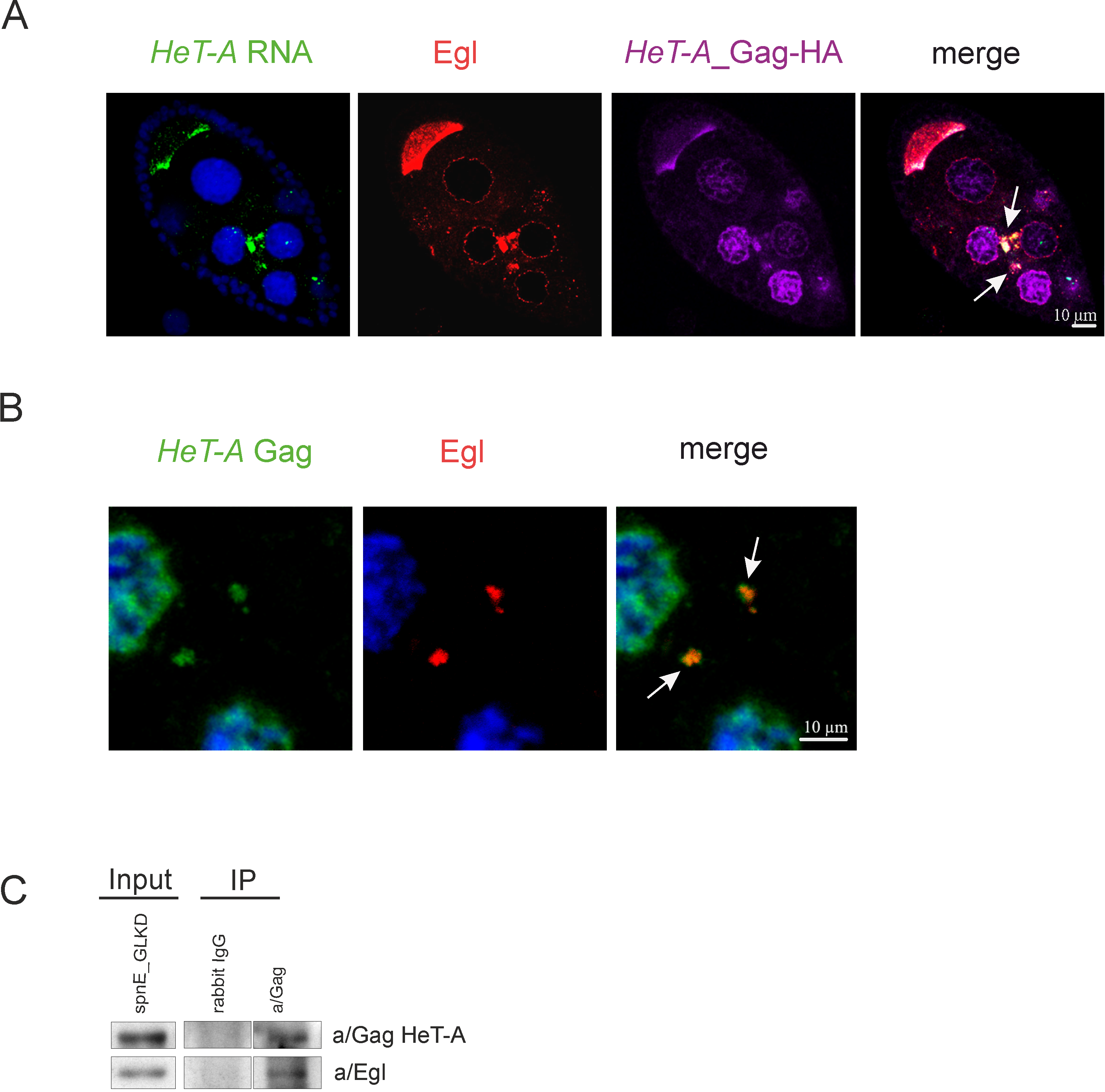

Supplement: S5 Fig — (A) HeT-A RNA (green), HeT-A Gag-HA (magenta) and Egl (red) form granules (arrows) in the cytoplasm of nurse cells in ovaries of nosGal4; UAS-HeT-A-HA; UAS-spnE_sh flies. Egg chamber at stage 7 of oogenesis is shown. (B) Egl (red) and endogenous HeT-A Gag (green) form granules (arrows) in ovaries of spnE_GLKD not carrying UAS-HeT-A-HA transgene. DNA is stained with DAPI (blue). (C) Co-IP of HeT-A Gag. Western blot analysis of proteins immunoprecipitated with anti-Gag HeT-A from ovaries of spnE_GLKD flies. Anti-Gag HeT-A immunoprecipitates Egl protein. The antibodies used for Western blotting are indicated to the right and the antibodies used for co-IP are indicated above the IP lanes. (TIF) [file pone.0201787.s005.tif]

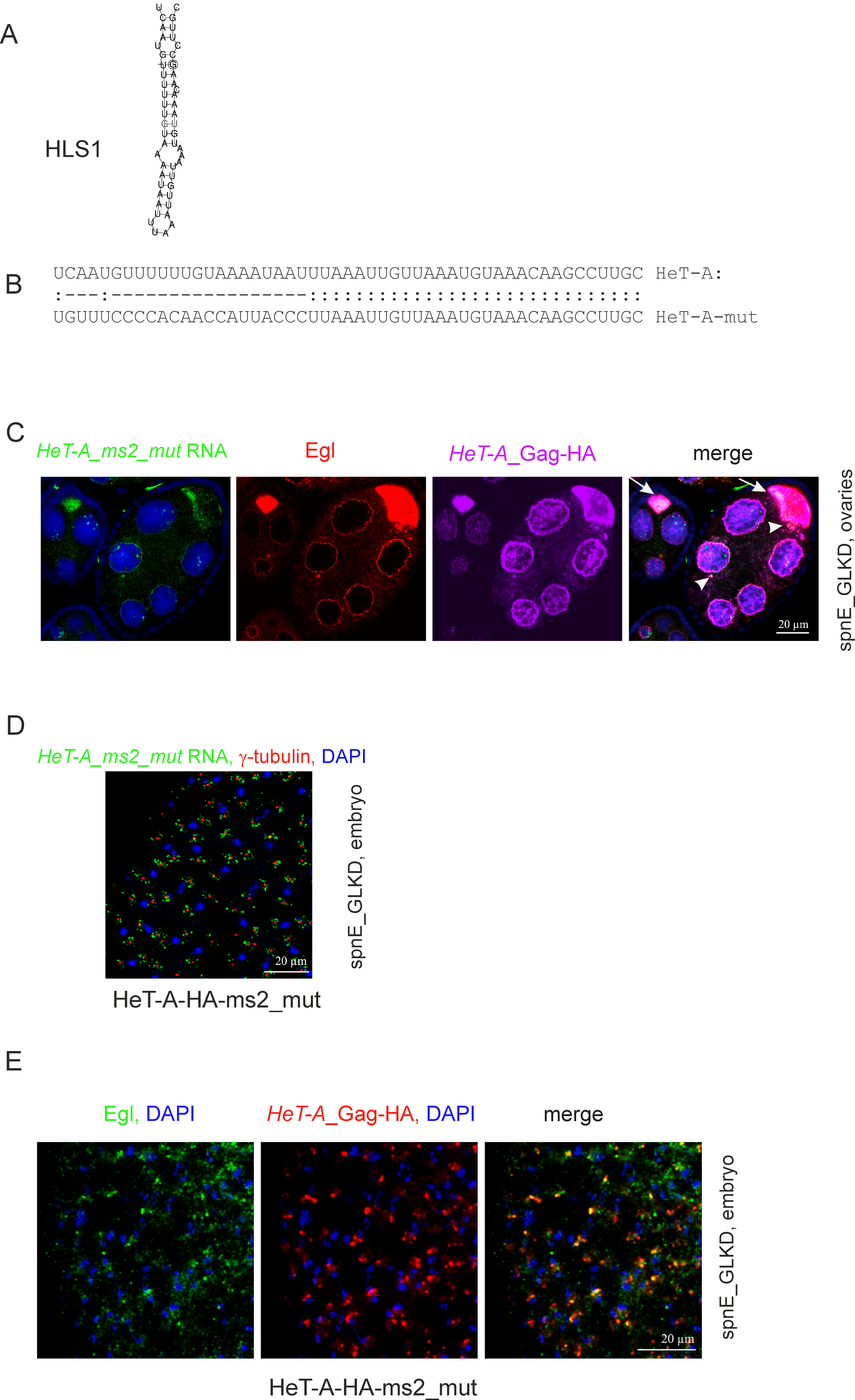

Supplement: S6 Fig — (A) A putative HLS1 (HeT-A localization signal 1) is revealed in the 3’UTR of HeT-A copies (start corresponds to 5798 position of canonical HeT-A, DM06920). Folding of HLS1 is shown. (B) Sequence of mutated HeT-A hairpin is shown. (C) Colocalization of MS2 RNA (green), HeT-A Gag-HA (magenta) and Egl (red) in the cytoplasm of nurse cells (arrowheads) and in the oocyte (arrows) in ovaries of nosGal4; UAS-HeT-A-HA-ms2_mut flies upon spnE_GLKD. Two egg chambers at different stages of oogenesis are shown. (D) MS2 RNA FISH (green) combined with immunostaining of gamma-tubulin (red) was performed on 0-2-hour old embryos of nosGal4; UAS-HeT-A-HA-ms2_mut flies upon spnE_GLKD. DNA is stained with DAPI (blue). Syncytial metaphase is shown. (E) HeT-A Gag-HA (red) and Egl (green) immunostaining were performed on 0-2-hour old embryos of nosGal4; UAS-HeT-A-HA-ms2_mut flies upon spnE_GLKD. DNA is stained with DAPI (blue). (TIF) [file pone.0201787.s006.tif]
